# Short-Term Metabolic Changes and Their Physiological Mediators in the Roux-en-Y Gastric Bypass Bariatric Surgery

**DOI:** 10.1007/s11695-023-07042-y

**Published:** 2024-01-09

**Authors:** Siyu Zhao, Sohvi Hörkkö, Markku J. Savolainen, Vesa Koivukangas, Ville-Petteri Mäkinen, Mika Ala-Korpela, Janne Hukkanen

**Affiliations:** 1 Systems Epidemiology, Research Unit of Population Health, Faculty of Medicine, University of Oulu, Oulu, Finland; 2 Research Unit of Population Health, Faculty of Medicine, University of Oulu, Oulu, Finland; 3 Biocenter Oulu, University of Oulu, Oulu, Finland; 4 Medical Microbiology and Immunology, Research Unit of Biomedicine, University of Oulu, Oulu, Finland; 5 Medical Research Center Oulu, Oulu University Hospital and University of Oulu, Oulu, Finland; 6 Research Unit of Internal Medicine, University of Oulu, Oulu, Finland; 7 Department of Surgery, Oulu University Hospital and University of Oulu, Oulu, Finland; 8 NMR Metabolomics Laboratory, School of Pharmacy, Faculty of Health Sciences, University of Eastern Finland, Kuopio, Finland

**Keywords:** Obesity, Diabetes, Bariatric surgery, Epidemiology, Metabolomics, Insulin

## Abstract

**Background:**

The Roux-en-Y gastric bypass (RYGB) is a common bariatric surgery to treat obesity. Its metabolic consequences are favourable and long-term clinical corollaries beneficial. However, detailed assessments of various affected metabolic pathways and their mediating physiological factors are scarce.

**Methods:**

We performed a clinical study with 30 RYGB patients in preoperative and 6-month postoperative visits. NMR metabolomics was applied to profiling of systemic metabolism via 80 molecular traits, representing core cardiometabolic pathways. Glucose, glycated haemoglobin (HbA1c), insulin, and apolipoprotein B-48 were measured with standard assays. Logistic regression models of the surgery effect were used for each metabolic measure and assessed individually for multiple mediating physiological factors.

**Results:**

Changes in insulin concentrations reflected those of BMI with robust decreases due to the surgery. Six months after the surgery, triglycerides, remnant cholesterol, and apolipoprotein B-100 were decreased −24%, −18%, and −14%, respectively. Lactate and glycoprotein acetyls, a systemic inflammation biomarker, decreased −16% and −9%, respectively. The concentrations of branched-chain (BCAA; leucine, isoleucine, and valine) and aromatic (phenylalanine and tyrosine) amino acids decreased after the surgery between −17% for tyrosine and −23% for leucine. Except for the most prominent metabolic changes observed for the BCAAs, all changes were almost completely mediated by weight change and insulin. Glucose and type 2 diabetes had clearly weaker effects on the metabolic changes.

**Conclusions:**

The comprehensive metabolic analyses indicate that weight loss and improved insulin sensitivity during the 6 months after the RYGB surgery are the key physiological outcomes mediating the short-term advantageous metabolic effects of RYGB.

The clinical study was registered at ClinicalTrials.gov as NCT01330251.

**Graphical Abstract:**

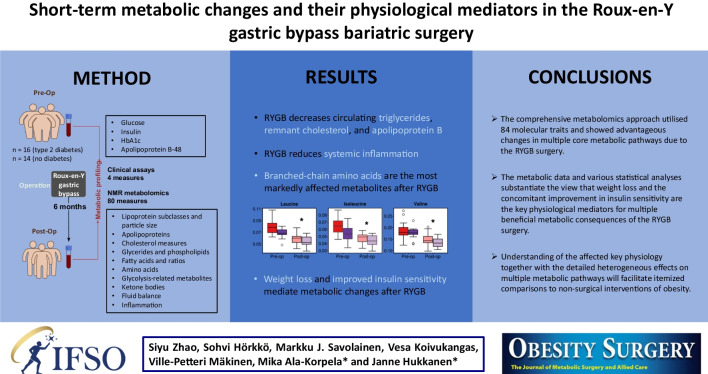

**Supplementary Information:**

The online version contains supplementary material available at 10.1007/s11695-023-07042-y.

## Introduction

Bariatric surgeries are a globally increasing trend to treat patients with obesity [1]. The key clinical reasons include evident long-term advantages on weight and appetite control, blood pressure, inflammation, mortality, liver health, as well as remission and prevention of type 2 diabetes [2–6]. Recent studies have demonstrated beneficial consequences of bariatric surgeries on multiple metabolic pathways [6–17]. However, limited attention has been paid on the potential mediating physiological factors explaining the abundant postoperative changes in circulating biomarkers [9–11, 16]. It is these physiological factors that are the primary result from the bariatric surgeries, not the metabolic changes per se. Thus, increased understanding of the metabolic effects of altered physiology is likely more important than just looking at how various metabolic biomarkers change [16]. Nonetheless, the comprehensive biomarker approach is a prerequisite to recognise the relative effects of resulting physiological changes on various metabolic pathways with potentially different clinical corollaries [6, 8–12, 14–16]. Understanding of the affected key physiology together with the detailed heterogeneous effects on multiple metabolic pathways will also facilitate itemised comparisons to non-surgical interventions of obesity [7, 16].

Here, we report a clinical study with 30 RYGB patients (16 with type 2 diabetes and 14 without) who completed the preoperative and 6-month postoperative study visits, including a comprehensive metabolic profiling of systemic metabolism. The 84 quantitative molecular traits, representing multiple core pathways in cardiometabolic health, facilitated wide-ranging analyses on the effects of weight loss and improved insulin sensitivity on the RYGB-induced systemic metabolic changes.

## Research Design and Methods

### Clinical Study Protocol and Patients

The study protocol has been described previously by Härma et al. [18], and more details are provided in the Supplementary Material. The study was registered at ClinicalTrials.gov as NCT01330251. Results are reported here for the 30 RYGB patients who completed both the pre- and postoperative study visits (16 with type 2 diabetes and 14 without). An outline of the study protocol with the metabolomic data characteristics is shown in Supplementary Fig. 1.

### Clinical Data

The use of medications was recorded at the study visits, and standard clinical measurements were performed. Insulin, glucose, and haemoglobin A1c (HbA1c) were determined by standard clinical assays. Apolipoprotein B-48 (apoB-48) was measured with an enzyme-linked immunosorbent assay. More details for the clinical measurements are given in the Supplementary Material. Clinical characteristics for the pre- and postoperative patients are given in Table 1.

**Table 1:** Clinical characteristics for the pre- and postoperative patients

Variable	No type 2 diabetes			With type 2 diabetes			All patients		
	Baseline	6 months	*p* value	Baseline	6 months	*P* value	Baseline	6 months	*p* value
Number of patients	14	14		16	16		30	30	
Age, years	44.5 ± 9.0			49.8 ± 8.1			47.3 ± 8.8		
Male, *n* (%)	3 (21)			6 (38)			9 (30)		
Hypertension, *n* (%)	8 (57)	-		13 (81)	-		21 (70)	-	
Weight, kg	133.3 ± 24.7	105.5 ± 23.9	0.005	120.5 ± 18.9	93.9 ± 13.2	6.7E−5	126.5 ± 22.4	99.3 ± 19.5	5.3E−4
Change from baseline weight, kg		−27.8 ± 7.0			−26.6 ± 8.6			−27.2 ± 7.8	
Total weight loss, %		−21 ± 6			−22 ± 4			-22 ± 5	
BMI, kg/m^2^	47.5 ± 5.6	37.4 ± 5.9	8.6E-5	41.8 ± 4.3	32.6 ± 3.2	1.5E−7	44.5 ± 5.7	34.8 ± 5.1	4.8E−9
Systolic BP, mmHg	127.9 ± 10.6	125.2 ± 9.2	0.5	130.3 ± 17.2	125.6 ± 14.7	0.4	129.2 ± 14.3	125.4 ± 12.3	0.3
Diastolic BP, mmHg	83.1 ± 6.6	78.2 ± 8.2	0.1	82.7 ± 9.4	76.8 ± 9.9	0.1	82.9 ± 8.1	77.5 ± 9.0	0.02
Fasting glucose, mmol/L	5.7 ± 0.5	5.3 ± 0.5	0.02	6.9 ± 1.4	6.0 ± 1.1	0.06	6.3 ± 1.2	5.7 ± 1.0	0.02
Fasting insulin, uM/L	22.4 ± 13.4	8.6 ± 3.4	9.7E−4	21.0 ± 14.6	10.4 ± 4.0	0.009	21.6 ± 13.8	9.6 ± 3.8	2.4E−5
HbA1c, mmol/mol	37.9 ± 3.3	36.6 ± 4.3	0.38	46.3 ± 6.1	40.7 ± 6.6	0.02	42.4 ± 6.5	38.8 ± 5.9	0.03
ApoB-48, ng/L	1201 ± 371	1459 ± 362	0.08	1249 ± 457	1519 ± 501	0.1	1227 ± 412	1491 ± 436	0.02

The data are presented as mean ± standard deviation. Ten subjects were on statin medication at the baseline visit; four of them had stopped the medication before the 6-month visit. Sixteen subjects had oral antidiabetic medications at baseline visit; all of them had stopped the medications before the 6-month visit

*BMI* body mass index, *BP* blood pressure, *ApoB-48* apolipoprotein B-48

### Metabolomic Data

A high-throughput nuclear magnetic resonance (NMR) metabolomic platform was applied [19, 20]. The 80 molecular outputs analysed feature 14 lipoprotein subclasses [21], apolipoprotein A-I (apoA-I) and B (apoB), multiple clinical cholesterol and triglyceride measures, albumin, various fatty acids, and numerous low-molecular-weight metabolites, including amino acids, glycolysis related measures, ketone bodies and a new inflammation marker glycoprotein acetyls (GlycA)—most of them in central pathways related to cardiometabolic health (Supplementary Fig. 1). The lipoprotein subclass data are described in detail in the Supplementary Material. The platform has been used in numerous epidemiological and genetic studies over the past 10 years, and it has also been adopted by the UK Biobank [19–22].

### Statistical Analyses

To manage multiple testing over the large set of metabolic measures, we first conducted principal component analysis to determine the effective number of independent variables. Sixteen principal components were enough to explain >95% of variation in the metabolic data. Therefore, we set the 5% Bonferroni-adjusted type 1 error threshold at *p* < 0.05/16 = 0.0031.

Logistic regression models of the surgery effect were constructed for each metabolic measure to manage potentially confounding factors such as sex and age as well as to analyse if the metabolic changes would be mediated by physiological co-variation because of the surgery. The dependent variable was set to 0 for the preoperative and 1 for the postoperative samples. Various models, namely sex + age, sex + age + BMI, sex + age + insulin, sex + age + glucose, and sex + age + history of type 2 diabetes, were tested to assess how the physiological co-variation between the pre- and postoperative time points would affect the metabolic changes. Before modelling, the inputs were scaled to unit standard deviation.

All analyses were undertaken on the R statistical platform (version 3.6.2).

## Results

Only two metabolites showed a different response to the RYGB operation between the patients with and without type 2 diabetes: glucose (*p* = 0.8*10^−3^) and alanine (*p* = 0.001) decreased more for those patients that had type 2 diabetes in the pre-RYGB time point. Thus, all the metabolic data were analysed and presented as combining these groups pre- and post-operation.

### BMI, Insulin, Glucose and HbA1c

**Fig. 1:**
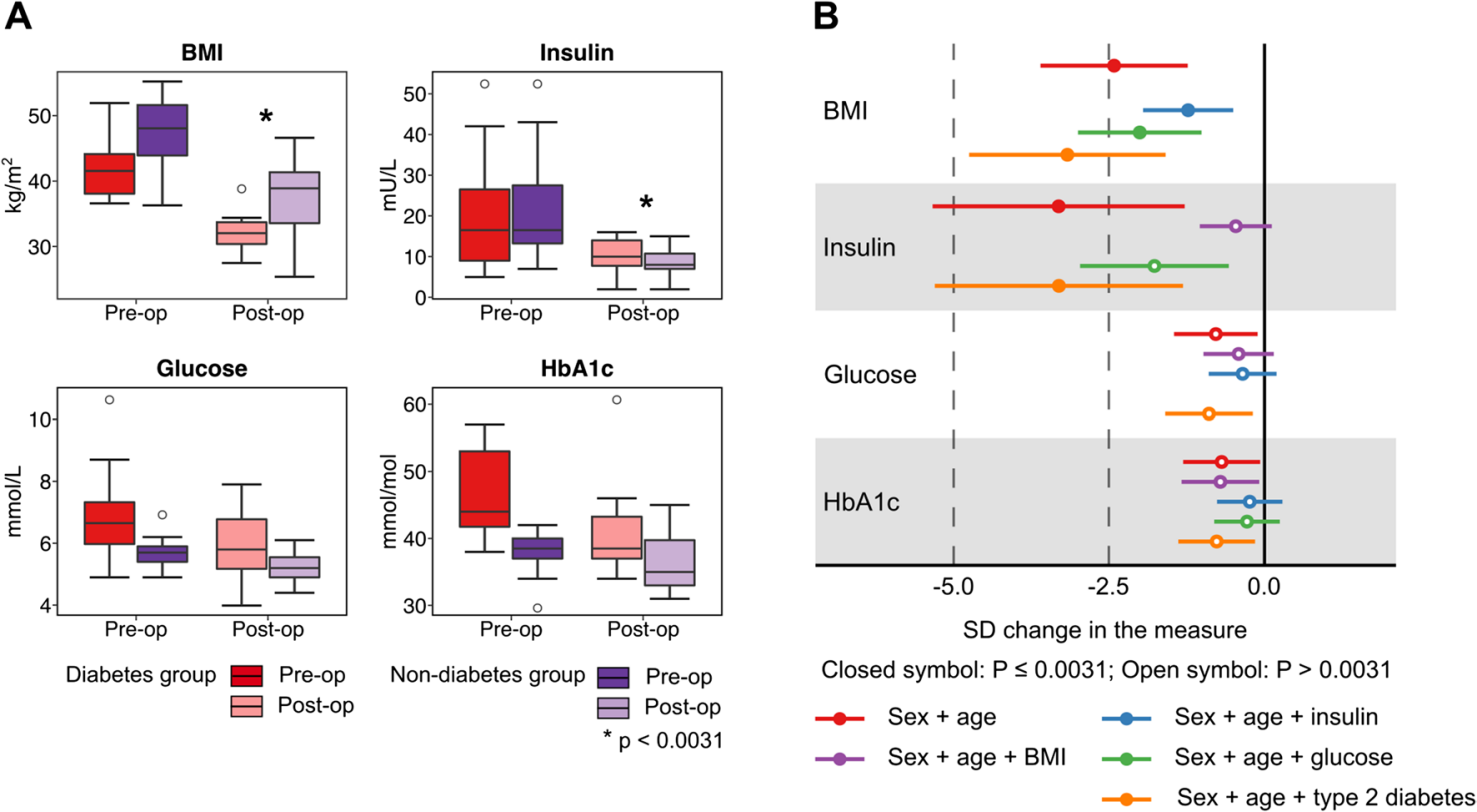
**A** Preoperative and 6-month postoperative distributions of BMI, insulin, glucose, and HbA1c. The box plots represent medians with interquartile ranges and with 10th percentile minimum and 90th percentile maximum whiskers. Open circles refer to outliers. Metabolite concentrations were compared using the paired *t* test. *Robust association for all the RYGB patients (*n* = 30) (i.e. including those who had and did not have type 2 diabetes) at the Bonferroni-corrected threshold *p* < 0.0031. **B** Regression modelling on changes in BMI, insulin, glucose, and HbA1c from the preoperative visit to the 6-month postoperative visit. Analyses with individual adjustments for sex + age, sex + age + BMI, sex + age + insulin, sex + age + glucose, and sex + age + type 2 diabetes are shown

The pre- and postoperative BMI ranges were 36.3 kg/m^2^ ≤ BMI ≤ 55.2 kg/m^2^ and 25.4 kg/m^2^ ≤ BMI ≤ 46.6 kg/m^2^, respectively. The expected, clear reduction in BMI 6 months after the RYBG surgery for both patient groups is shown in Fig. 1A; for the type 2 diabetes group (*n* = 16) from (median (interquartile range)) 41.55 (38.08–44.12) kg/m^2^ to 32.05 (30.40–33.73) kg/m^2^ and for the nondiabetic group (*n* = 14) from 48.05 (43.90–51.60) kg/m^2^ to 38.90 (33.58–41.35) kg/m^2^. Changes in insulin concentrations reflect those of BMI with robust decreases from 16.5 (13.3–27.5) mU/L to 8.0 (7.0–10.8) mU/L for the type 2 diabetes group and from 16.5 (9.0–26.5) mU/L to 10.0 (7.8–14.0) mU/L for the nondiabetic group. For both glucose and HbA1c, similar trends are seen in the median values, however with marked overlap between the pre- and postoperative distributions.

### Apolipoprotein B, Triglyceride, Cholesterol and Lipoprotein Subclass Particle Concentrations

**Fig. 2:**
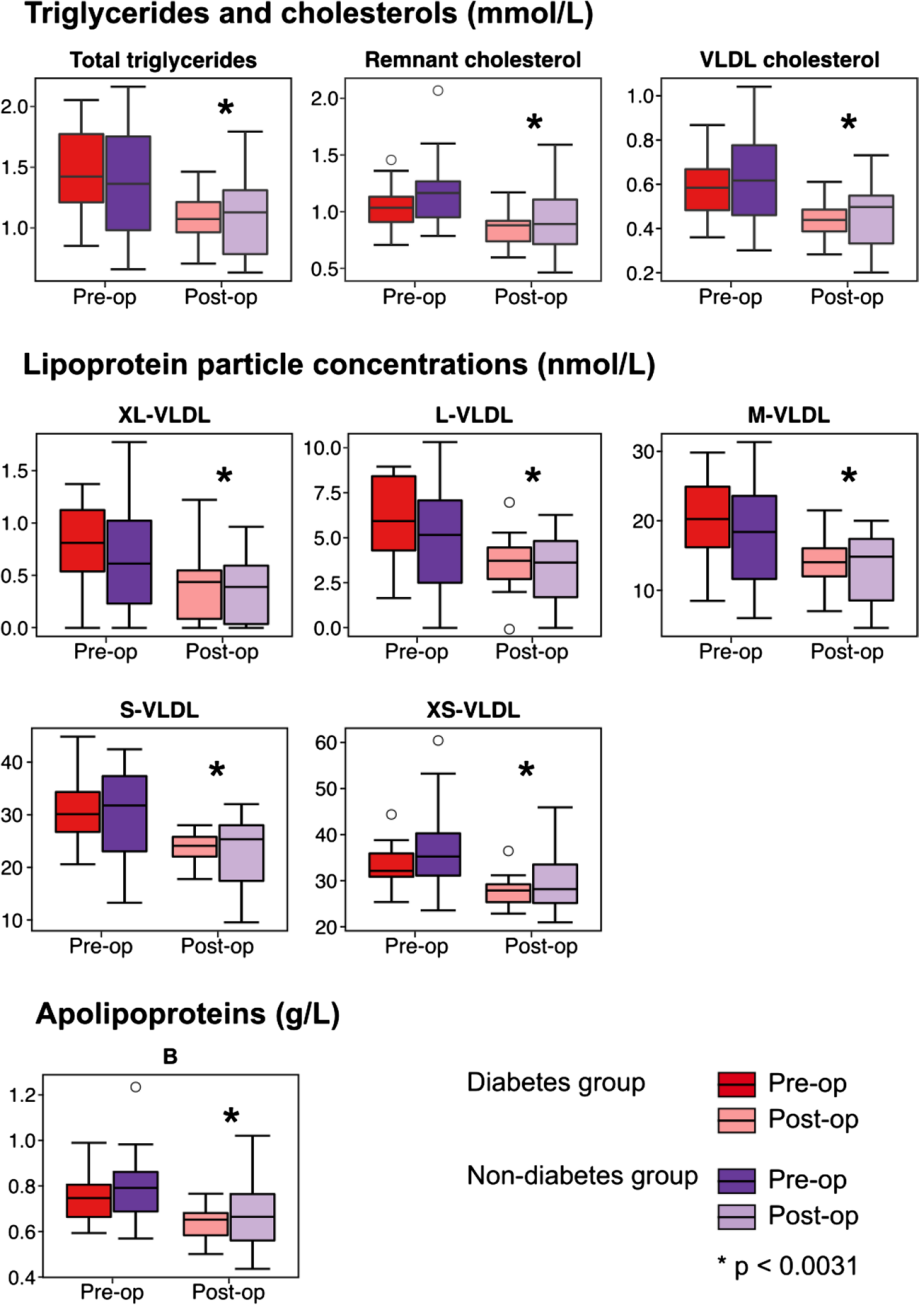
Preoperative and 6-month postoperative distributions for various lipoprotein measures. The box plots represent medians with interquartile ranges and with 10th percentile minimum and 90th percentile maximum whiskers. Open circles refer to outliers. Metabolite concentrations were compared using the paired *t* test. *Robust association for all the RYGB patients (*n* = 30) (i.e. including those who had and did not have type 2 diabetes) at the Bonferroni-corrected threshold *p* < 0.0031

Serum triglycerides, VLDL cholesterol, and remnant cholesterol (i.e. non-HDL, non-LDL cholesterol [23]) show robust decreases of around −24%, −26%, and −18%, respectively, after the surgery (Fig. 2). The lipoprotein subclass data indicates that all the VLDL subclass particle concentrations follow a similar robust trend of decrease. Circulating apoB concentrations show a robust decrease of around −14% after the RYGB surgery, reflecting the trends of all the individual apoB-containing lipoprotein subclass particles (Fig. 2 and Supplementary Fig. 2).

### Fatty Acids and Amino Acids

**Fig. 3:**
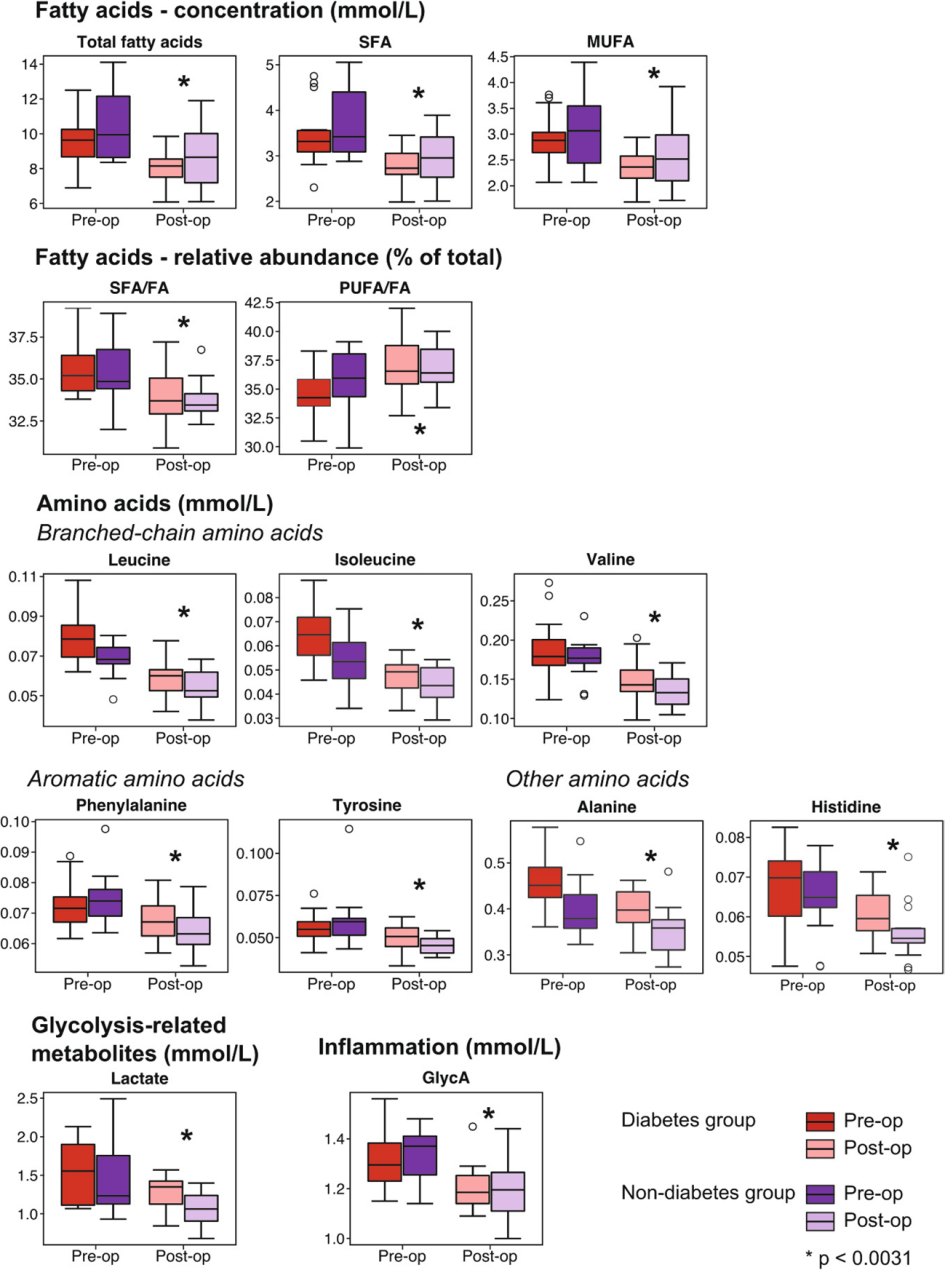
Preoperative and 6-month postoperative distributions for various fatty acid concentrations and relative abundances. Corresponding distributions are also shown for seven circulating amino acid concentrations, lactate, and the inflammation marker GlycA. The box plots represent medians with interquartile ranges and with 10th percentile minimum and 90th percentile maximum whiskers. Open circles refer to outliers. Metabolite concentrations were compared using a paired *t* test. *Robust association for all the RYGB patients (*n* = 30) (i.e. including those who had and did not have type 2 diabetes) at the Bonferroni-corrected threshold *p* < 0.0031

The concentrations of total (−17%) as well as saturated (SFA, −16%) and monounsaturated (MUFA, −19%) fatty acids were robustly decreased after the operation for both patient groups as illustrated in Fig. 3. In relative terms of total fatty acids, the proportion of SFA robustly decreases similar to the decrease in the absolute concentration. The proportion of PUFAs shows a robust increase in contradiction to the decreasing trend in absolute concentrations (Supplementary Fig. 2).

The concentrations of branched-chain amino acids (BCAAs; leucine, isoleucine, and valine), aromatic amino acids (phenylalanine and tyrosine), as well as histidine and alanine show robust decreases 6 months after the RYGB surgery (Fig. 3). The decrease varies between −17% for tyrosine and −23% for leucine.

### Glycolysis-Related Metabolites and Inflammation

Lactate and GlycA, a recently emerged inflammation biomarker [24], show a robust decrease of −16% and −9%, respectively, after the surgery (Fig. 3).

The results for the metabolic distributions and their changes after the RYGB surgery, corresponding to Figs. 1A, 2, and 3, for all the 84 metabolic measures plus BMI are illustrated in Supplementary Fig. 2.

### Regression Models

Figure 1B depicts results from regression modelling, together with the effects of various adjustments (sex + age, sex + age + BMI, sex + age + insulin, sex + age + glucose, and sex + age + history of type 2 diabetes), on how BMI, insulin, glucose, and HbA1c changed between the 6-month time point after the RYGB surgery and the preoperative time point. The BMI change is not explained by adjusting for insulin or glucose changes, but the effect of the insulin adjustment is larger. The adjustment for BMI abolishes and the adjustment for glucose have a marked effect on the change in insulin concentrations. The associations for glucose and HbA1c are weak in all the models. Adjusting for type 2 diabetes has very little effect on the associations for all the clinical variables. It should be noted that all the patients using oral antidiabetic medications before RYGB had stopped the medications prior to the 6-month visit.

**Fig. 4:**
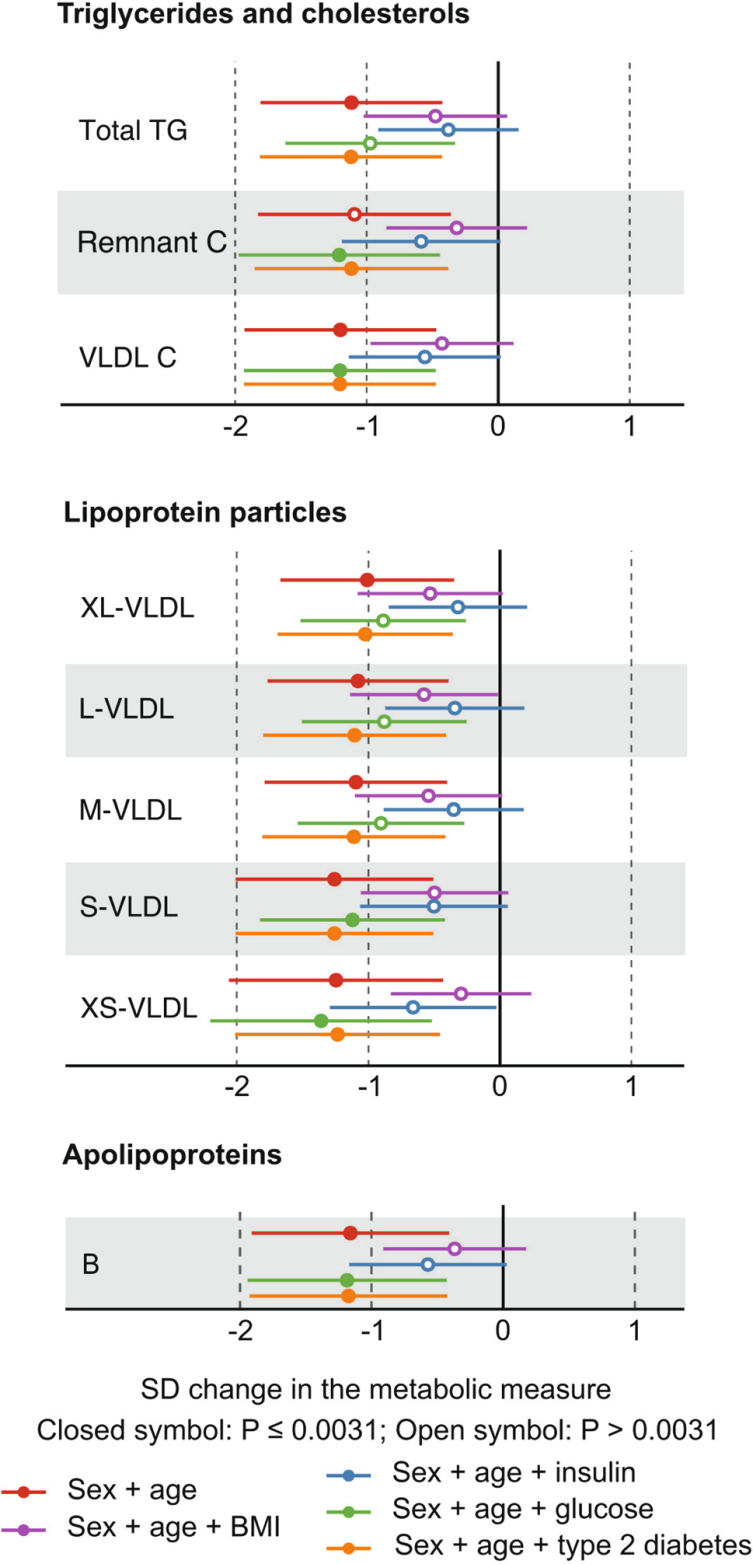
Regression modelling on metabolic changes in the concentrations of various lipoprotein measures from the preoperative visit to the 6-month postoperative visit. Analyses with individual adjustments for sex + age, sex + age + BMI, sex + age + insulin, sex + age + glucose, and sex + age + type 2 diabetes are shown

**Fig. 5:**
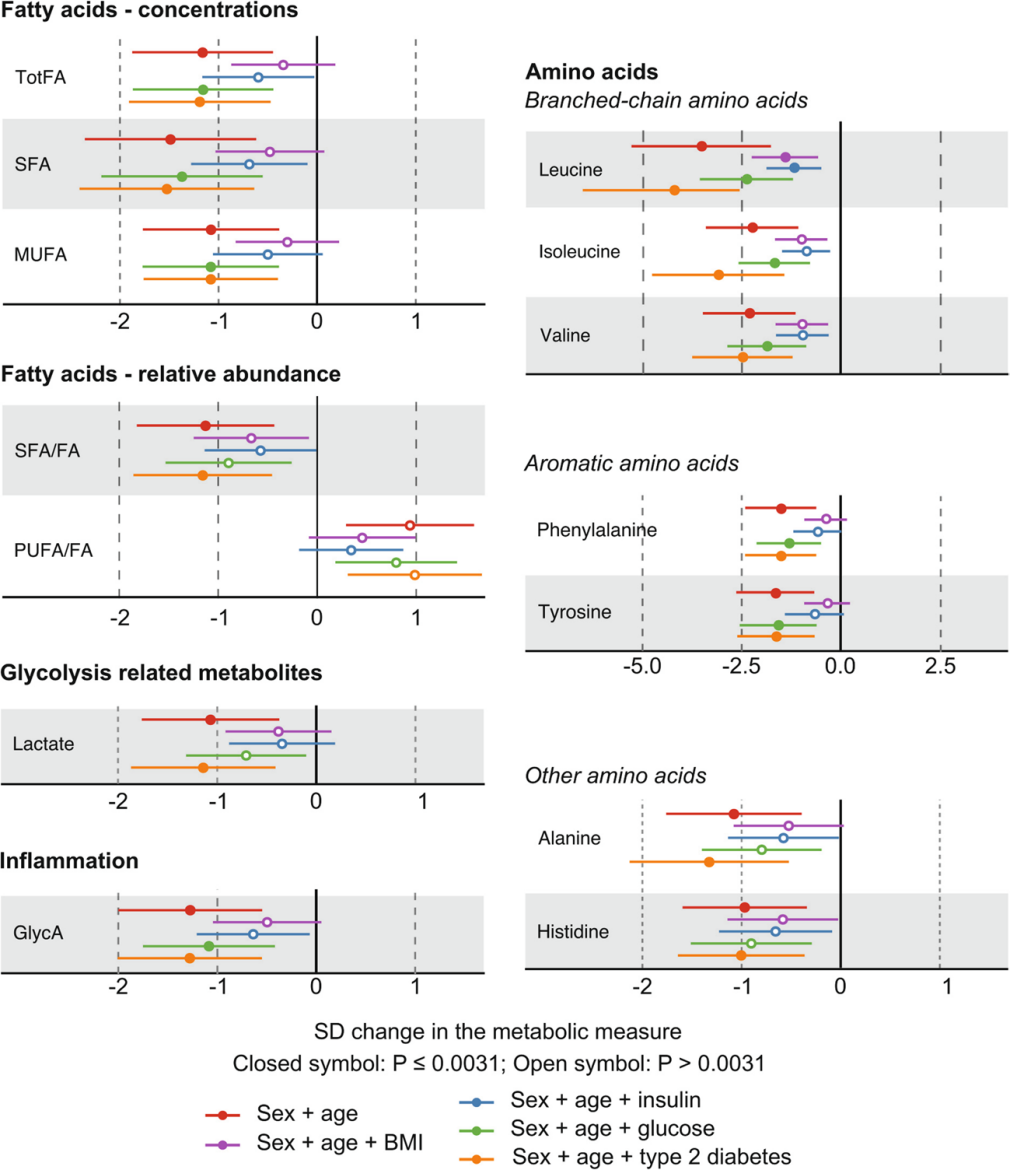
Regression modelling on metabolic changes in various fatty acid concentrations and relative abundances from the preoperative visit to the 6-month postoperative visit. Corresponding analyses are also shown for seven circulating amino acid concentrations, lactate, and the inflammation marker GlycA. Analyses with individual adjustments for sex and age, sex and age + BMI, sex and age + insulin, sex and age + glucose, and sex and age + type 2 diabetes are shown

The results from the abovementioned regression modelling are illustrated for the various lipid, lipoprotein, and apolipoprotein measures in Fig. 4. Overall, both BMI and insulin change have strong effects on the models, and the effects of both glucose change and type 2 diabetes are minor. Very similar results are seen for the fatty acids, amino acids, glycolysis-related metabolites, and the inflammation biomarker GlycA (Fig. 5). The associations for the changes in the BCAAs are prominent and remain robust after adjusting for glucose change. Moreover, they are not abolished after adjusting for BMI and insulin. The associations for leucine are slightly stronger than those for isoleucine and valine.

The results for the regression modelling, corresponding to Figs. 1B, 4, and 5, for all the 84 metabolic measures and BMI are illustrated in Supplementary Fig. 3.

## Discussion

This study focused on the 6-month postoperative changes in the systemic metabolism of patients who went through the Roux-en-Y gastric bypass surgery to treat morbid obesity. The clinical characteristics and outcomes were as expected with a substantial decrease in BMI and markedly improved insulin sensitivity (Fig. 1). The systemic metabolomic approach, utilising 84 quantitative molecular traits, enabled detection of accompanying characteristic changes in multiple core metabolic pathways related to cardiometabolic health. The current results replicate many known metabolic consequences of RYGB alongside providing more nuance, for example, for the lipoprotein subclass sequels, inflammation, amino acids, glycolysis-related measures, and ketone bodies (Figs. 2 and 3). We also showed that the ample postoperative metabolic changes along diverse metabolic pathways are, to a great degree, mediated by the weight loss and by the concomitant increase in insulin sensitivity, but not by changes in circulating glucose concentrations (Figs. 4 and 5).

The characteristic effects of the RYGB surgery on lipoprotein and lipid metabolism are well-known at the level of standard clinical lipid measures [5]. The primary effect was, as expected, a clear decrease in the circulating triglyceride concentrations. The extensive lipoprotein data acquired here illustrate that this effect is coherently seen in the circulating particle concentrations of all the VLDL subclasses and reflected by the decreases in remnant and VLDL cholesterol as well as in the circulating apoB concentrations. Our results are broadly similar to those in a recent study that used another NMR-based method to analyse changes in detailed lipoprotein profiles due to bariatric surgeries [17]. As a logical result of decreased concentrations of circulating triglycerides, all the circulating concentrations of fatty acids showed a decreasing trend after the RYGB surgery. Alongside these absolute decreases in concentrations, the relative amount of saturated fatty acids decreased and that of polyunsaturated fatty acids increased.

Most literature related to circulating fatty acids in RYGB focuses particularly on non-esterified fatty acids, i.e. free fatty acids (FFAs) [10, 15]. The fatty acid measures here from the NMR metabolomics platform include all fatty acids in the serum samples, and therefore, these measures mostly reflect the average fatty acid composition in all the major lipid classes in all the circulating lipoprotein particles with only minor contributions from lipid molecules not transported in the lipoprotein particles, e.g. those bound to albumin [19, 20]. However, while being summary measures, they are associated with insulin resistance [25] and more generally with cardiometabolic risk [26] although detailed comparisons to studies in which only FFAs have been analysed are not feasible.

Branched-chain amino acids have been studied extensively in relation to obesity and type 2 diabetes [25] as well as to the RYBG and other bariatric procedures [15, 27, 28]. Our new findings are compatible with previous results and illustrate that all BCAA concentrations are markedly decreased after the RYBG operation for patients with and without type 2 diabetes. Consistent decreasing trends were also noted for the aromatic amino acids (phenylalanine and tyrosine) as well as for alanine. All these amino acid findings conform to multiple previous studies as reviewed by Vaz et al. [15]. Previous studies have reported inconsistent findings for the circulating concentrations of histidine after bariatric surgery, but here, with the RYGB procedure, the histidine concentration was decreased. The inconsistencies with histidine may relate to dissimilarities in the various types of bariatric surgery [15, 27].

Concurrent with previous results for the C-reactive protein (CRP) [3], a recently introduced systemic inflammation biomarker, glycoprotein acetyls [24], also decreased after the RYGB surgery. GlycA is a heterogeneous biomarker associated with both acute and chronic inflammation, and it provides partly CRP-independent information on various disease risks and frailty [29]. The current result thus adds support for systemic anti-inflammatory effects after the RYGB surgery. However, similarly to CRP [3], changes in circulating GlycA concentrations relate strongly to changes in insulin sensitivity.

For those metabolic measures that robustly changed after the RYGB surgery, the typical change was in the order of one SD from the baseline value. This was the case for the lipoprotein subclasses and lipids, most fatty acids and the amino acids alanine and histidine. The magnitude of change was also around one SD for glucose and HbA1c, but these associations were not robust. Slightly larger changes were seen for the saturated fatty acid concentration, GlycA and the aromatic amino acids phenylalanine and tyrosine. The largest magnitudes were around 2.5 times baseline SD for BMI, insulin, and BCAAs. These degrees of changes after the RYGB surgery are in the ballpark of metabolic changes detected in longitudinal observational studies in relation to changes in BMI, i.e. typical changes in metabolic measures are between 0.1 and 0.2 SD per BMI-unit [30].

Almost all the above-mentioned metabolic changes after the RYGB surgery were statistically explained by changes in BMI and insulin. In contradiction, adjustments for changes in circulating glucose concentrations had only minor or no effects. The same was the case for adjusting for the type 2 diabetes status, suggesting that the metabolic effects of the RYGB surgery were similar for all the patients with obesity whether they did or did not have type 2 diabetes before the operation. Previous metabolomic studies of RYGB, and bariatric surgeries in general, have paid limited attention on the potential factors explaining the abundant postoperative metabolic changes [9–11, 16]. The coherent role of weight loss [16] and insulin in explaining changes over diverse metabolic pathways, evident from the current results, is in accordance with the known concomitant increase of insulin sensitivity along the long-term weight loss after RYGB [31]. The genetic background for insulin resistance is highly pleiotropic with effects on lipoprotein metabolism, peripheral adipose tissue characteristics, and risk of cardiometabolic diseases [32]. Recent genetic analyses on the causal effects of insulin resistance on the systemic metabolism also give explicit support on the key role of insulin in explaining the metabolic findings [33].

The current analyses also pinpointed pronounced decreases in the circulating concentrations of alanine and lactate after the RYGB surgery, the associations being also strongly affected by the change in insulin sensitivity. Nonetheless, the abovementioned genetic causality analyses for insulin resistance substantiated neither of these circulating metabolites [33]. High circulating alanine and lactate levels associate with liver fat [34] and with non-optimal functioning of hypoxia-inducible factors (HIFs) [35], the key regulators of oxygen homeostasis in response to hypoxia. HIFs are known to be repressed in diabetic conditions [36]. Thus, while insulin resistance is likely associated with all these interrelated phenomena, their relative contributions for circulating alanine and lactate concentrations might be heavily overlapped making the dissection of the underlying physiological origins problematic [37].

Wide-ranging metabolic studies on the effects of bariatric surgeries have started to accumulate only recently [7–16, 38]. The strengths of this work are the comprehensive metabolic and statistical analyses that substantiate the view that weight loss and the concomitant improvement in insulin sensitivity are the key physiological mediators for multiple beneficial metabolic consequences of the RYGB surgery. This study, as most bariatric surgery studies, is limited by the number of patients and thus has statistical limitations to detect minor changes due to the RYGB operation. Nonetheless, robust metabolic changes were observed for multiple molecular pathways, and the statistical power for the regression modelling on the mediating physiological factors was good.

### Supplementary Information

ESM 1 (DOCX 8.84 MB)

## Data Availability

The data for the clinical RYGB study are available from the corresponding authors upon reasonable request.
