# Supplementary material for: Short-Term Metabolic Changes and Their Physiological Mediators in the Roux-en-Y Gastric Bypass Bariatric Surgery
Source: Obes Surg. 2024 Jan 9;34(2):625–34. doi: 10.1007/s11695-023-07042-y (PMC10810963; doi:10.1007/s11695-023-07042-y)
Supplement: Supplementary file 1 — (DOCX 8.84 MB) [file 11695_2023_7042_MOESM1_ESM.docx]

# **Short-term metabolic changes and their physiological mediators in the Roux-en-Y gastric bypass bariatric surgery**

Siyu Zhao, Sohvi Hörkkö, Markku J. Savolainen, Vesa Koivukangas, Ville-Petteri Mäkinen, Mika Ala-Korpela, Janne Hukkanen

# **SUPPLEMENTARY MATERIAL**

**Clinical Study Protocol and Patients**

**Lipoprotein subclass data**

**Figure S1.** Outline of the study protocol with the metabolomics data characteristics.

**Figure S2.** Preoperative and 6-months postoperative distributions in the clinical RYGB study for all the 84 metabolic measures plus BMI.

**Figure S3.** Regression modelling on the differences between the 6-month postoperative and the preoperative time point in the clinical RYGB study for all the 84 metabolic measures plus BMI.

**Clinical Study Protocol and Patients**

We recruited individuals with a clinically indicated need for a bariatric surgery (1). The inclusion criteria were a medical indication for bariatric surgery and an age between 18 and 65 years. The indications for RYGB in patients without T2DM were 1) BMI > 40, or 2) BMI > 35 and an additional obesity-related comorbidity such as hypertension, sleep apnoea, osteoarthritis of major joints, PCOS, or reflux disease. For patients with T2DM, BMI limit is lower (> 30). The exclusion criteria were a need for insulin therapy (type 1 or type 2 diabetes) or oral corticosteroids; a history of prolonged antibiotic treatments; chronic inflammatory diseases, including IBD and rheumatic diseases; coeliac disease; and malignant diseases.

The first study visit before the surgery was designed to occur before initiating the preoperative very-low-calorie (VLC) diet. However, 6 participants (4 with type 2 diabetes and 2 without) had started the VLC diet already before their first study visit due to scheduling difficulties. All procedures performed in studies involving human participants were in accordance with the ethical standards of the institutional and national research committee and followed the ethical standards of the Helsinki Declaration, as revised in 2000. The study protocol was approved by the Ethics Committee of the Northern Ostrobothnia Hospital District. All patients provided written informed consent before any study-related procedure. The study was registered at ClinicalTrials.gov as NCT01330251.

The inclusion criteria were a medical indication for bariatric surgery and an age between 18 and 65 years. The exclusion criteria were a need for insulin therapy (type 1 or type 2 diabetes) or oral corticosteroids; a history of prolonged antibiotic treatments; chronic inflammatory diseases, including inflammatory bowel disease and rheumatic diseases; coeliac disease; and malignant diseases. The study was terminated due to slow recruitment when a total of 34 patients had been enrolled. There were 3 individuals who declined the 6-month study visit and 1 individual who declined surgery after the first study visit.

Weight was measured with a Seca 861 scale (Seca GmbH & Co., Hamburg, Germany), and body mass index was calculated as weight divided by height squared (kg/m2). In addition, insulin, glucose, and HbA1c were assessed by standard clinical assays. Apolipoprotein B-48 was measured with an enzyme-linked immunosorbent assay according to the instructions of the manufacturer (Human ApoB48 ELISA kit; Elabscience, Wuhan, China).

All blood samples were taken after an overnight fast. Data on dietary details were not available.

**Lipoprotein subclass data**

The separation of lipoprotein subclasses by proton NMR spectroscopy is based on particle size (2). The NMR data in this particular platform are calibrated via high-performance liquid chromatography with the platform resolution being 14 lipoprotein subclasses, defined by their particle size as follows: six very-low-density lipoprotein (VLDL) particle categories [extremely large (XXL-VLDL, with average particle diameter >75 nm), very large (XL-VLDL, 64 nm), large (L-VLDL, 53.6 nm), medium (M-VLDL, 44.5 nm), small (S-VLDL, 36.8 nm) and very small (XS-VLDL, 31.3 nm)]; intermediate-density lipoprotein particles (IDL, 28.6 nm), three LDL particle categories [large (L-LDL, 25.5 nm), medium (M-LDL, 23.0 nm) and small (S-LDL, 18.7 nm)]; and four HDL particle categories [very large (XL-HDL, 14.3 nm), large (L-HDL, 12.1 nm), medium (M-HDL, 10.9 nm) and small (S-HDL, 8.7 nm)] (3,4). Independent verification for the robust NMR resolution with respect to the number of lipoprotein subclasses was published by Mihaleva and co-workers with an in-depth handling of the statistical grounds (5).

**References**

1. Härma MA, Adeshara K, Istomin N, Lehto M, Blaut M, Savolainen MJ, et al. Gastrointestinal manifestations after Roux-en-Y gastric bypass surgery in individuals with and without type 2 diabetes. Surg Obes Relat Dis Off J Am Soc Bariatr Surg. 2021 Mar;17(3):585–94.

2. Ala-Korpela M. 1H NMR spectroscopy of human blood plasma. Prog Nucl Magn Reson Spectrosc [Internet]. 1995 Nov 1 [cited 2020 Oct 19];27(5):475–554. Available from: http://www.sciencedirect.com/science/article/pii/0079656595010130

3. Soininen P, Kangas AJ, Würtz P, Suna T, Ala-Korpela M. Quantitative serum nuclear magnetic resonance metabolomics in cardiovascular epidemiology and genetics. Circ Cardiovasc Genet. 2015 Feb;8(1):192–206.

4. Ala-Korpela M, Zhao S, Järvelin MR, Mäkinen VP, Ohukainen P. Apt interpretation of comprehensive lipoprotein data in large-scale epidemiology: disclosure of fundamental structural and metabolic relationships. Int J Epidemiol [Internet]. 2021 Aug 18 [cited 2021 Aug 18];(dyab156). Available from: https://doi.org/10.1093/ije/dyab156

5. Mihaleva VV, van Schalkwijk DB, de Graaf AA, van Duynhoven J, van Dorsten FA, Vervoort J, et al. A systematic approach to obtain validated partial least square models for predicting lipoprotein subclasses from serum NMR spectra. Anal Chem. 2014 Jan 7;86(1):543–50.

**Figure S1.** Outline of the clinical study protocol with the metabolomics data characteristics. Abbreviations: BMI, body mass index; Pre-Op, preoperative; Post-Op, postoperative; HbA1c, haemoglobin A1c; VLDL, very-low-density lipoprotein; IDL, intermediate-density lipoprotein; LDL, low-density lipoprotein; HDL, high-density lipoprotein; XXL, extremely large; XL, extra large; L, large; M, medium; S, small; XS, extra small; TG, triglycerides; C, cholesterol; PC, phosphatidylcholine (and other cholines); SM, sphingomyelins; FA, fatty acids; PUFA, polyunsaturated FA; MUFA, monounsaturated FA; SFA, saturated FA; DHA, docosahexaenoic acid; LA, linoleic acid; GlycA, glycoprotein acetyls.

*Continued on the next page*

*
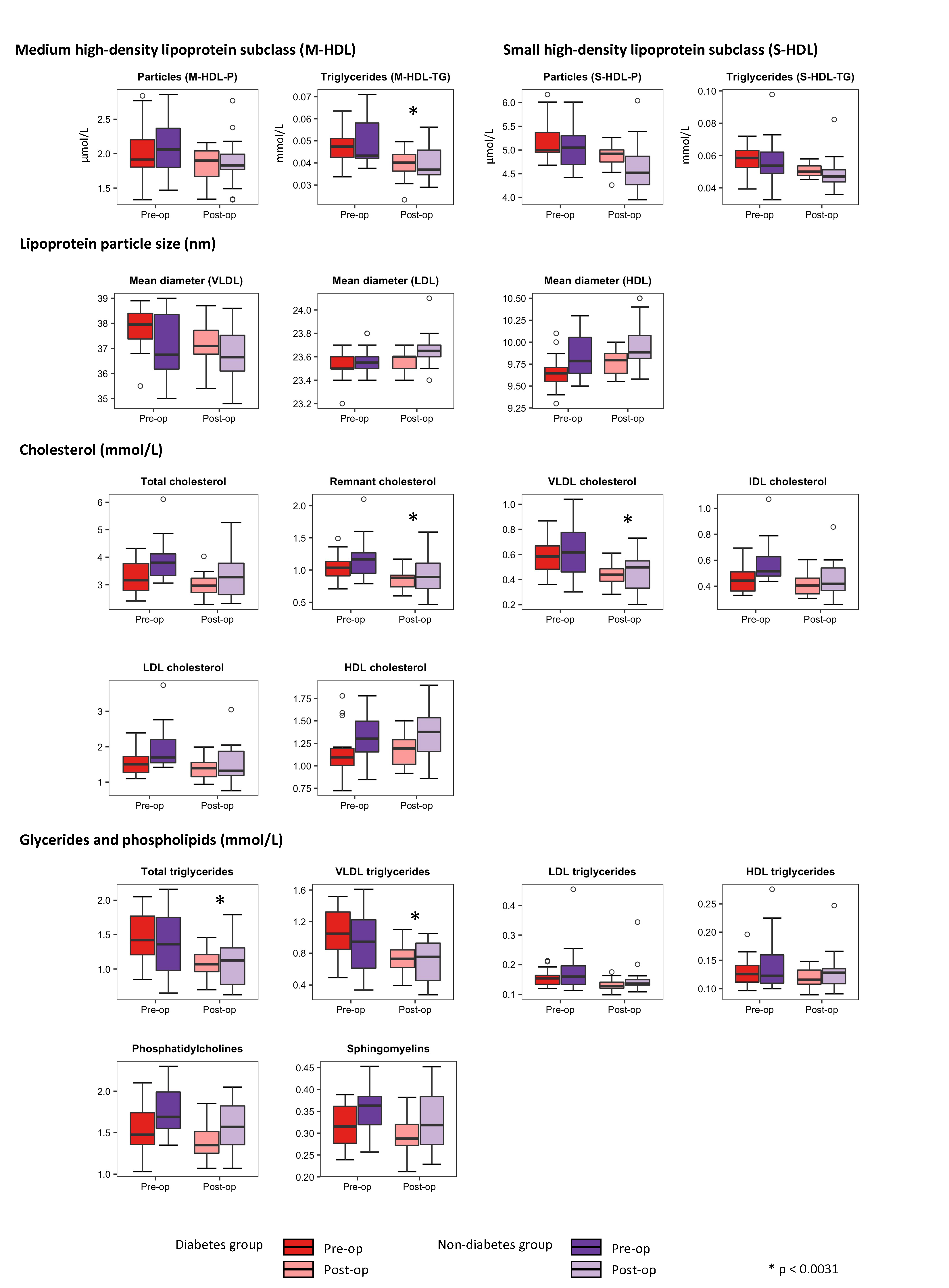
*

*Continued on the next page*

*
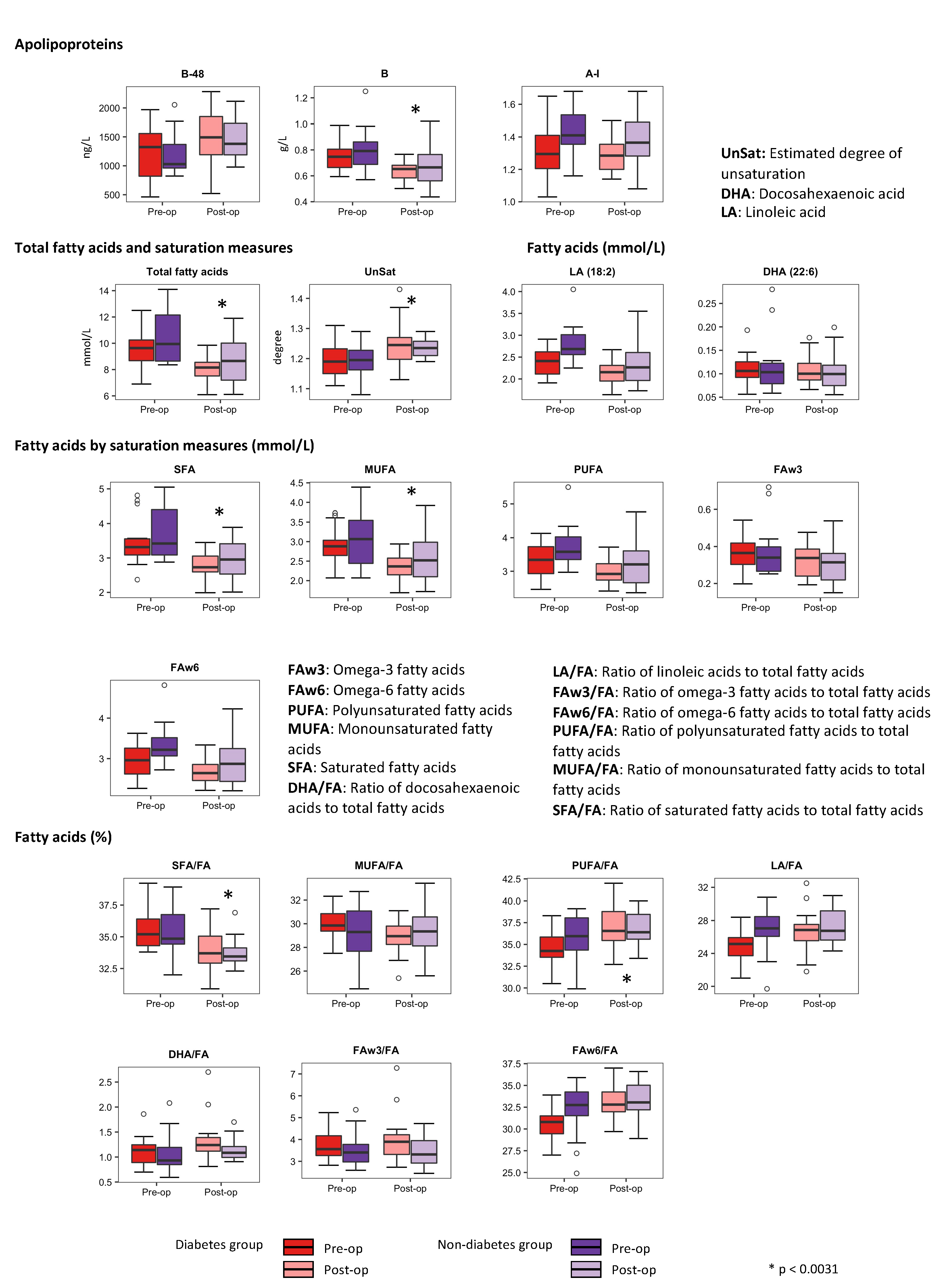
Continued on the next page*

**Figure S2.** Preoperative and 6-month postoperative distributions in the clinical RYGB study for all the 84 metabolic measures plus BMI. The box plots represent medians with interquartile ranges and with 10^th^ percentile minimum and 90^th^ percentile maximum whiskers. Open circles refer to outliers. Metabolite concentrations were compared using a paired t-test. *Robust association for all the RYGB patients (n = 30) (i.e., including those who had and did not have type 2 diabetes) at the Bonferroni-corrected threshold p < 0.0031.


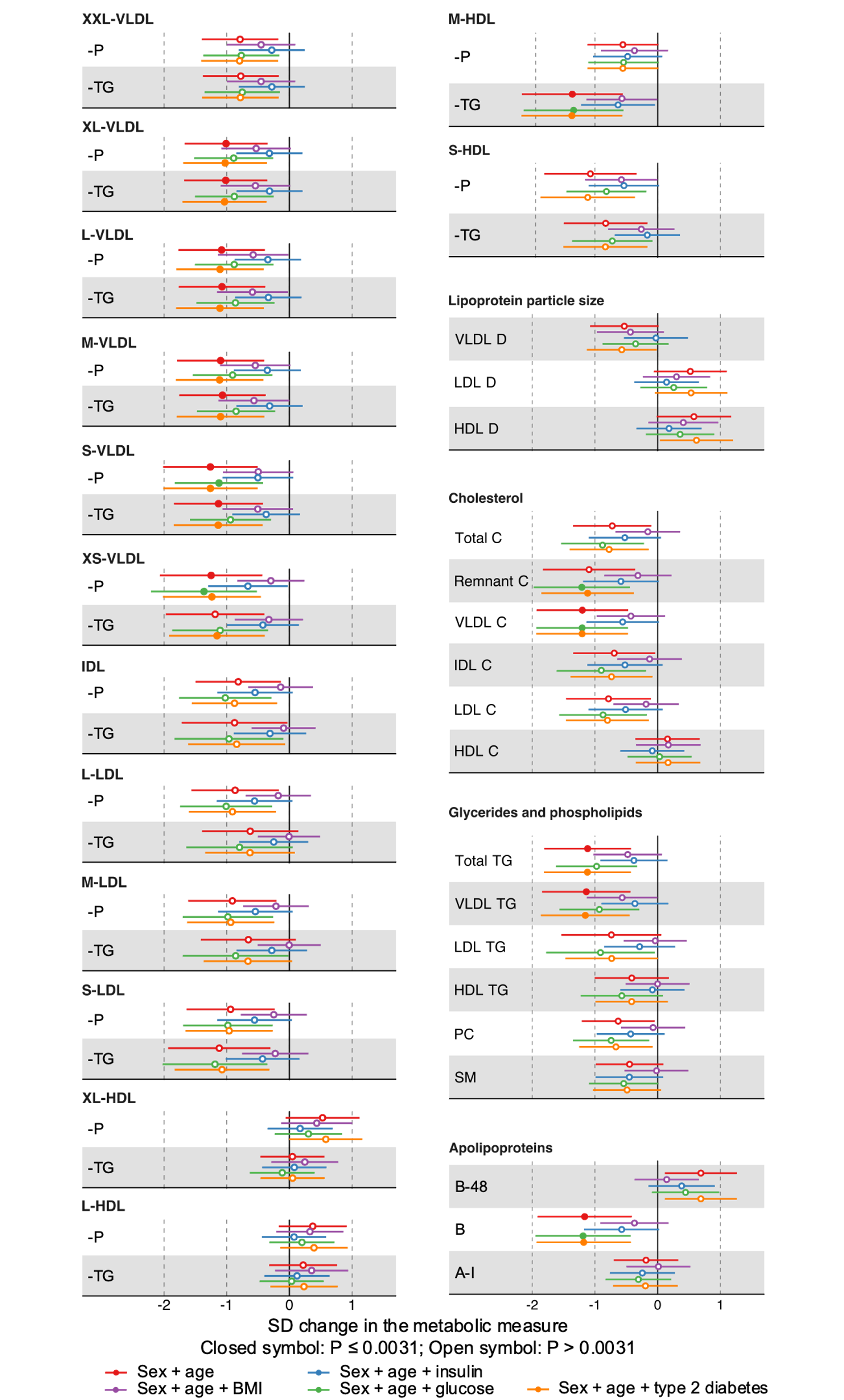


*Continued on the next page*


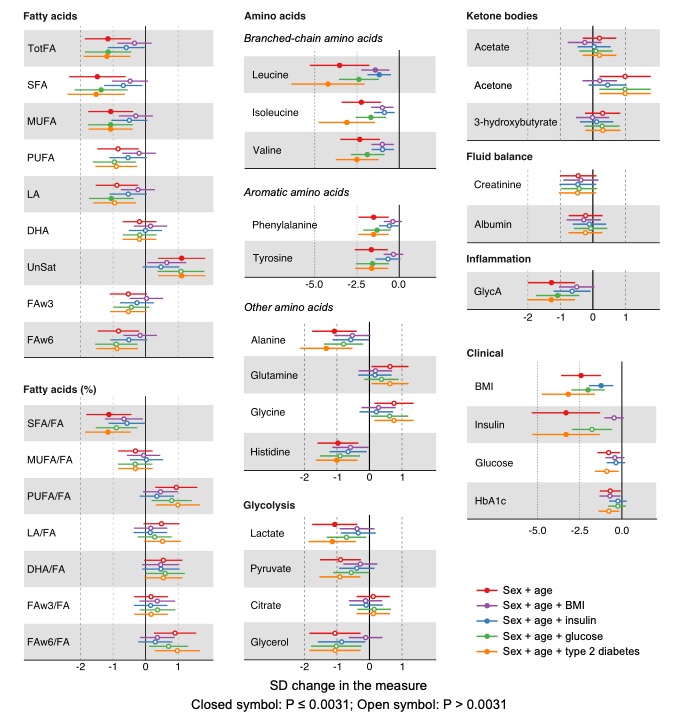


**Figure S3.** Regression modelling of the changes from preoperative visit to the six-month postoperative visit in the clinical RYGB study for all the 84 metabolic measures plus BMI. Analyses with individual adjustments for sex + age, sex + age + BMI, sex + age + insulin, sex + age + glucose, and sex + age + type 2 diabetes are shown.
